# The critical role that STAT3 plays in glioma-initiating cells: STAT3 addiction in glioma

**DOI:** 10.18632/oncotarget.25188

**Published:** 2018-04-24

**Authors:** Debolina Ganguly, Meiyun Fan, Chuan He Yang, Blazej Zbytek, David Finkelstein, Martine F. Roussel, Lawrence M. Pfeffer

**Affiliations:** ^1^ Department of Pathology and Laboratory Medicine, and Center for Cancer Research, University of Tennessee Health Science Center, Memphis, TN, USA; ^2^ Pathology Group of the Midsouth, Germantown, TN, USA; ^3^ Department of Computational Biology, St. Jude Children’s Research Hospital, Memphis, TN, USA; ^4^ Department of Tumor Cell Biology, St. Jude Children’s Research Hospital, Memphis, TN, USA

**Keywords:** STAT3, glioblastoma, phosphorylation, gene expression, tumorigenesis

## Abstract

Glioma-Initiating Cells (GICs) are thought to be responsible for tumor initiation, progression and recurrence in glioblastoma (GBM). In previous studies, we reported the constitutive phosphorylation of the STAT3 transcription factor in GICs derived from GBM patient-derived xenografts, and that STAT3 played a critical role in GBM tumorigenesis. In this study, we show that CRISPR/Cas9-mediated deletion of STAT3 in an established GBM cell line markedly inhibited tumorigenesis by intracranial injection but had little effect on cell proliferation *in vitro*. Tumorigenesis was rescued by the enforced expression of wild-type STAT3 in cells lacking STAT3. In contrast, GICs were highly addicted to STAT3 and upon STAT3 deletion GICs were non-viable. Moreover, we found that STAT3 was constitutively activated in GICs by phosphorylation on both tyrosine (Y705) and serine (S727) residues. Therefore, to study STAT3 function in GICs we established an inducible system to knockdown STAT3 expression (iSTAT3-KD). Using this approach, we demonstrated that Y705-STAT3 phosphorylation was critical and indispensable for GIC-induced tumor formation. Both phosphorylation sites in STAT3 promoted GIC proliferation *in vitro*. We further showed that S727-STAT3 phosphorylation was Y705-dependent. Targeted microarray and RNA sequencing revealed that STAT3 activated cell-cycle regulator genes, and downregulated genes involved in the interferon response, the hypoxia response, the TGFβ pathway, and remodeling of the extracellular matrix. Since STAT3 is an important oncogenic driver of GBM, the identification of these STAT3 regulated pathways in GICs will inform the development of better targeted therapies against STAT3 in GBM and other cancers.

## INTRODUCTION

Glioblastoma (GBM; World Health Organization Grade IV glioma) is the most common and deadliest brain cancer in adults. Despite advances in surgery and therapy, the median survival of patients is only between 10-15 months [1, 2]. The highly aggressive and diffuse infiltrative nature of GBM makes it extremely difficult to treat. Furthermore, the rapid tumor recurrence after surgical resection and treatment by radiotherapy and chemotherapy demonstrates the ability of the residual tumor cells to evade treatment and propagate. Tumor recurrence and therapeutic resistance has been attributed to Glioma-Initiating Cells (GICs) within the tumor that display several characteristics of neural stem cells [2–4]. The high tumor-initiating capabilities of the GICs, and their ability to evade therapy and differentiate into multiple cell types suggest that GICs contribute to GBM maintenance and tumor relapse. Therefore, identifying pathways critical for GIC function is essential for developing new strategies to target GICs, and to improve GBM patient survival.

STAT3 is a member of the Signal Transducer and Activator of Transcription (STAT) family of transcription factors, which is constitutively activated in various cancers, including GBM. STAT3 promotes tumor growth and angiogenesis, inhibits immune responses, and promotes tumor invasion and metastasis [5–7]. STAT3 contains a DNA binding domain and a C-terminal transactivation domain which undergoes both Tyrosine (Y) 705 and Serine (S) 727 phosphorylation, which regulate STAT3 activity. Many cytokines, growth factors and G-protein coupled receptors induce Y705-STAT3 phosphorylation [7, 8], demonstrating that diverse pathways lead to constitutive STAT3 activity in cancer. Upon Y705 phosphorylation, STAT3 homodimerizes and/or heterodimerizes with other STAT proteins, and translocates into the nucleus to regulate gene transcription. The role of S727 phosphorylation is not well understood although evidence indicates that it may be required for maximum transcriptional activity [9]. Previously, we reported that STAT3 undergoes constitutive Y705 phosphorylation in GICs isolated from several GBM patient-derived xenograft (PDX) models [10, 11]. In addition, we also found that a STAT3 inhibitor (WP1066) prevented STAT3 Y705 phosphorylation and attenuated GIC-driven tumor growth [11].

In the present study, we showed that STAT3 deletion in an established GBM cell line inhibited tumorigenesis *in vivo*, but it had little effect on cell proliferation *in vitro*. In contrast, STAT3 deletion in GICs resulted in a loss of cell viability. In order to study STAT3 function, we developed a Doxycycline (Dox)-inducible STAT3 knockdown (KD) (iSTAT3-KD) system in GICs to determine whether the STAT3 Y705 and S727 phosphorylation sites play distinct roles in regulating GIC function *in vitro* and *in vivo*. We found that STAT3 is critical for GBM tumorigenesis and that the Y705 phosphorylation site is indispensable for GIC tumor growth. Moreover, S727-STAT3 phosphorylation also plays an important role in GIC function *in vitro*. By targeted arrays and RNA-sequence analysis, we found STAT3 regulated genes in GICs that are important in various pro-tumorigenic pathways.

## RESULTS

### Deletion of STAT3 inhibits GBM tumorigenesis

**Figure 1:**
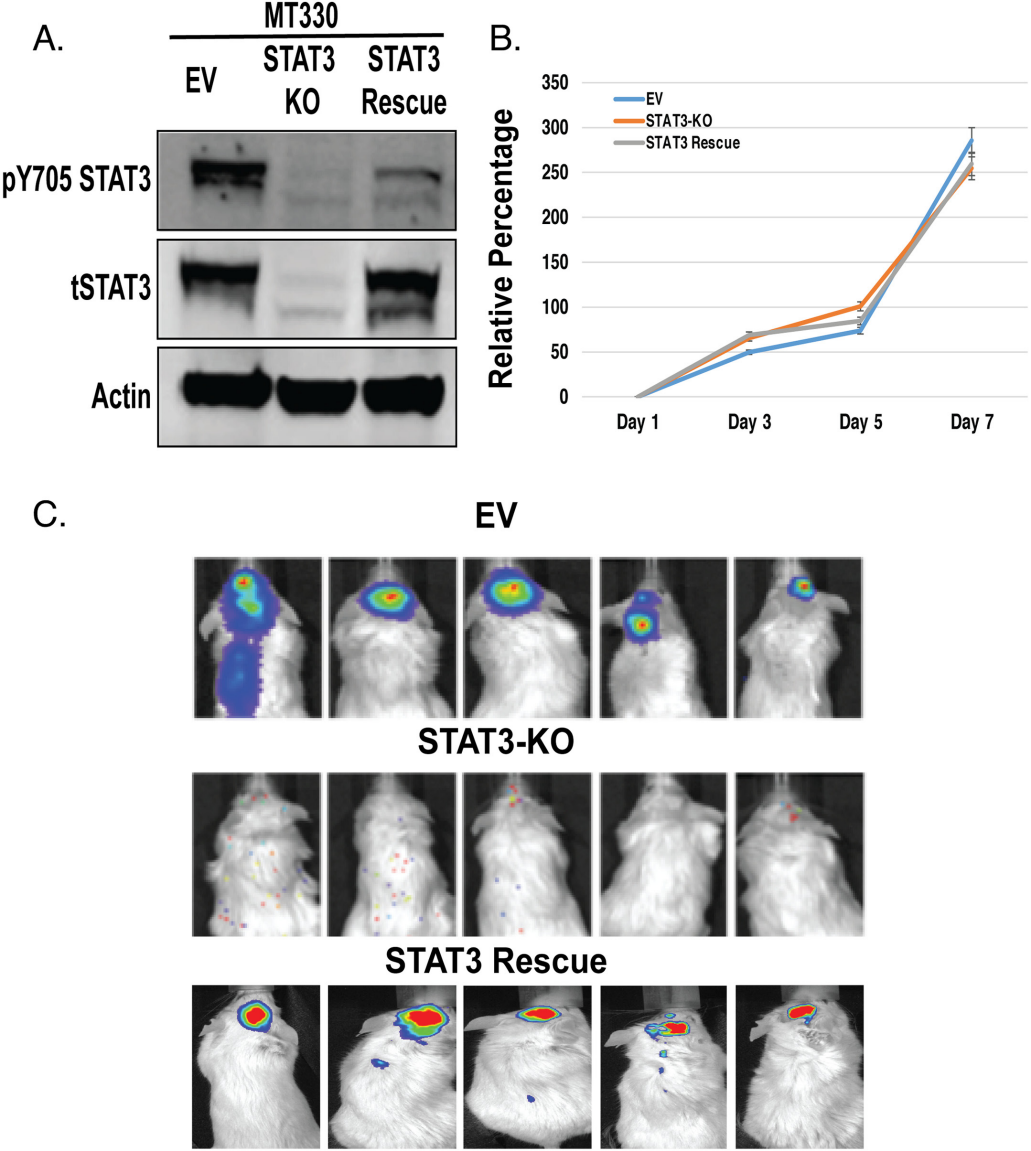
Role of STAT3 on MT330 GBM cell proliferation and tumorigenicity MT330 cells were transduced with empty vector (EV), STAT3 was deleted by CRISPR/Cas9 gene editing (STAT3-KO cells) and STAT3-KO cells were rescued with enforced expression of WT-STAT3. **(A)** Cell lysates were analyzed by immunoblotting for pY705-STAT3 and total STAT3. **(B)** Cell proliferation was determined CellTiter-Glo assays. **(C)** Tumorigenicity was assessed by injection of 10^6^ tumor cells into the brains of NSG mice and live animal imaging was performed at 21 days post-injection.

To investigate the role of STAT3 in GBM, we deleted the *STAT3* gene in MT330 GBM cells by CRISPR/Cas9 gene editing. As control, MT330 cells were transduced with empty vector (EV). STAT3 loss (STAT3-KO) was validated in whole cell extracts by immunoblotting with antibodies to STAT3 (Figure 1A). Deletion of STAT3 and rescue with wild type (WT)-STAT3 had no significant effect on the proliferation of these cells *in vitro* (Figure 1B). To assess tumorigenic potential, cells were injected into the brains of immunocompromised NSG mice, and tumor development was monitored by live animal imaging. While MT330 cells expressing an empty vector (EV MT330) formed brain tumors, loss of STAT3 markedly inhibited tumor formation (Figure 1C). Furthermore, STAT3 expression in STAT3-KO MT330 cells restored the ability of orthotopically-injected cells to form tumors (Figure 1C). Taken together, these results demonstrate that STAT3 plays a critical role in GBM tumorigenesis, but not in the proliferation of a GBM cell line *in vitro*.

### GICs have enhanced serine and tyrosine phosphorylation of STAT3 when compared to differentiating GICs

**Figure 2:**
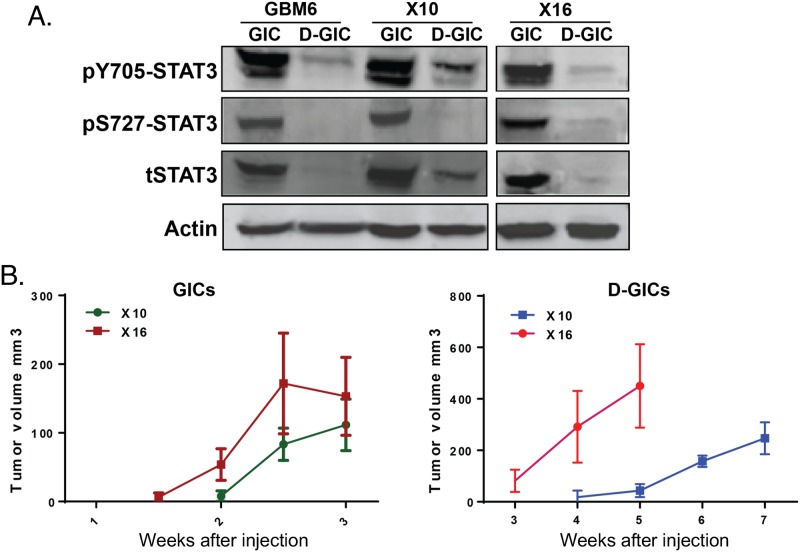
STAT3 phosphorylation and tumorigenicity of GICs and GICs induced to differentiate GICs were grown under stem cell conditions or induced to differentiate in the presence of serum. **(A)** Protein lysates were immunoblotted for pY705-STAT3, pS727-STAT3 and total STAT3. **(B)** Tumorigenicity was assessed by injection of 10^6^ tumor cells into the flanks of NSG mice and tumors were palpated every week.

Conventional GBM cell lines, like MT330 cells, grown in the presence of serum have been the mainstays of preclinical brain cancer research. However, the process of *in vitro* selection of established cell lines results in the irreversible loss of important properties, as they do not recapitulate the genomic and phenotypic properties of the original tumor [12, 13]. We and others found that GICs isolated from PDXs of surgical samples from GBM patients recapitulate the heterogeneity of GBM, and are responsible for the initiation, propagation and recurrence of GBM [10, 14]. We sought to determine STAT3 expression and phosphorylation in GICs isolated from three different PDXs, and in GICs induced to differentiate in the presence of serum (D-GICs). Differentiation was confirmed by increased protein expression of the astrocyte marker Glial Fibrillary Acidic Protein (GFAP), as well as the decreased expression of several neural stem cells markers, including *NESTIN* and *SOX2* (Supplementary Figure 1A, 1B). Levels of phosphorylated Y705 (pY705)-STAT3 and S727 (pS727)-STAT3, and total STAT3 protein were much higher in GICs isolated from GBM6, GBMX10 and GBMX16 PDXs as compared to their differentiating counterparts (Figure 2A). These results are consistent with our previous findings that pY705-STAT3 is significantly higher in GICs [15].

The tumorigenic potential of GBMX10 and GBMX16 GICs, and D-GICs was determined by injection into the flanks of NSG mice. Palpable masses were detected ∼2 weeks after injection of GBMX10 or GBMX16 GICs (Figure 2B). The experiments were terminated at 3 weeks, because mice started to show evidence of weight loss and physical distress. In contrast, tumors induced by D-GICs were first detected at 3-4 weeks after injection and mice survived up to 5-7 weeks after injection (Figure 2C). Furthermore, GIC-induced tumors formed and progressed much faster than the tumors produced by the D-GICs. These results are consistent with the hypothesis that GICs are primarily responsible for the initiation and progression of GBM tumors.

### Establishing an inducible STAT3 knockdown (iSTAT3-KD) system in GICs, and expression of the STAT3 phosphorylation-defective mutants

To explore the functional role of STAT3, we initially attempted to isolate STAT3-KO GICs by CRISPR/Cas9 gene editing. In contrast to the established MT330 GBM cells, GICs appeared to rely on STAT3 for proliferation and survival *in vitro*, since during puromycin selection the STAT3-KO GICs ceased to proliferate and became nonviable. Furthermore, constitutive knockdown (KD) of STAT3 expression in GICs with shRNA was also unsuccessful. However, a doxycycline (Dox)-inducible knockdown approach targeting STAT3 (iSTAT3-KD) with shRNA was highly effective. Both GBMX10 and GBMX16 GICs were transduced with the iSTAT3-KD construct that encoded red fluorescent protein (RFP), and stable pools of cells established by flow sorting for RFP. In order to investigate the functional role of pY705-STAT3 and pS727-STAT3, the RFP positive iSTAT3-KD GICs were transduced with a lentiviral vector encoding enhanced green fluorescent protein (E-GFP) and either the wild-type (WT)-STAT3 or the STAT3 phosphorylation-defective mutants (Y705F and S727A). All subsequent experiments were conducted with iSTAT3-KD GICs flow sorted for double-positive RFP (resulting in endogenous STAT3-KD) and GFP (resulting in exogenous STAT3 expression) cells.

**Figure 3:**
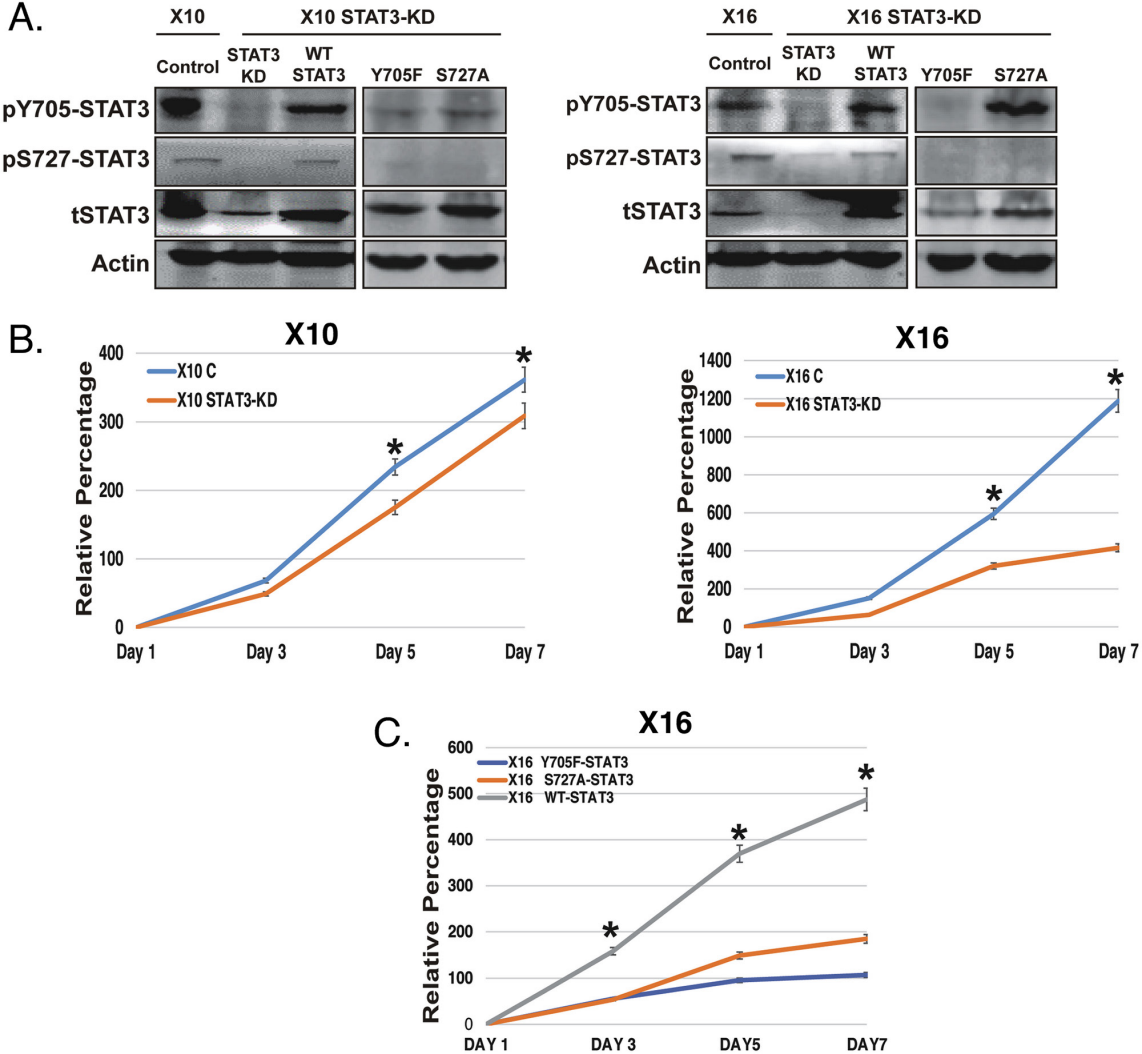
The effect of STAT3-KD and expression of STAT3 phosphorylation-defective mutants on STAT3 phosphorylation and GIC proliferation GBMX10 and GBMX16 GICs were transduced with a lentiviral vector containing a Dox-inducible shRNA against STAT3, and then transduced with wild type (WT) and mutant (Y705F and S727A) STAT3 constructs to restore STAT3 expression. **(A)** Cell lysates were analyzed by immunoblotting for pY705-STAT3, pS727-STAT3 and total STAT3. **(B)** Proliferation of control and STAT3-KD GBMX10 and GBMX16 GICs was determined CellTiter-Glo assays. **(C)** CellTiter-Glo based proliferation assays with the GBMX16 GICs harboring the STAT3 mutants and WT-STAT3 in the iSTAT3-KD background.

After Dox-treatment, GBMX10 and GBMX16 iSTAT3-KD GICs showed markedly reduced total STAT3, as well as reduced pS727 and pY705 STAT3 (Figure 3A). Expression of the mutant Y705F-STAT3 construct in iSTAT3-KD GICs not only ablated pY705-STAT3 but also led to loss of pS727-STAT3 (Figure 3A, 3B). However, expression of the mutant S727A-STAT3 construct in iSTAT3 KD-GICs had no effect on Y705-STAT3 phosphorylation, but blocked S727-STAT3 phosphorylation (Figure 3A). This finding indicates that Y705-STAT3 phosphorylation is required for S727-STAT3 phosphorylation to occur in GICs, but that S727-STAT3 phosphorylation is not required for Y705 phosphorylation. As expected, rescue with the WT-STAT3 construct in iSTAT3-KD GICs showed STAT3 phosphorylation at both Y705 and S727 sites (Figure 3A). The selectivity of iSTAT3-KD in GICs is evidenced by finding that STAT1 and STAT5 protein levels were not altered by STAT3-KD in GBMX16 GICs (Supplementary Figure 2).

### Effect of STAT3-KD and expression of STAT3 mutants on GIC proliferation

To define the functional consequence of STAT3-KD *in vitro*, as well as the effect of restoration with the various STAT3 constructs, we next examined GIC proliferation by the CellTiter-Glo assay, which quantifies the number of viable cells. STAT3-KD resulted in only a slight reduction in the proliferation of GBMX10 iSTAT3-KD GICs after Dox-treatment (Figure 3B). The effect of STAT3-KD in GBMX10 GICs is highly reminiscent of the marginal effect of STAT3-KO had on MT330 cell proliferation *in vitro* (Figure 1B). In contrast, STAT3-KD had a marked inhibitory effect on the proliferation of GBMX16 GICs (Figure 3B). Therefore, we examined the effects of STAT3 rescue with either WT-STAT3 or the STAT3 mutants on the proliferation of GBMX16 iSTAT3-KD GICs. Rescue of WT-STAT3 expression increased cell proliferation to that of GBMX16 GICs expressing STAT3, i.e. iSTAT3 KD-GICs without Dox-treatment (Figure 3C). In contrast, expression of Y705F-STAT3 did not rescue GBMX16 iSTAT3 KD-GIC proliferation, while expression of S727A-STAT3 only slightly rescued iSTAT3 KD-GIC proliferation (Figure 3C). However, although STAT3 plays a critical role in the proliferation of GBMX16 GICs, and both STAT3 phosphorylation sites regulate cell proliferation, STAT3 did not regulate the proliferation of GBMX10 GICs.

### Effect of STAT3-KD on GIC tumorigenicity

**Figure 4:**
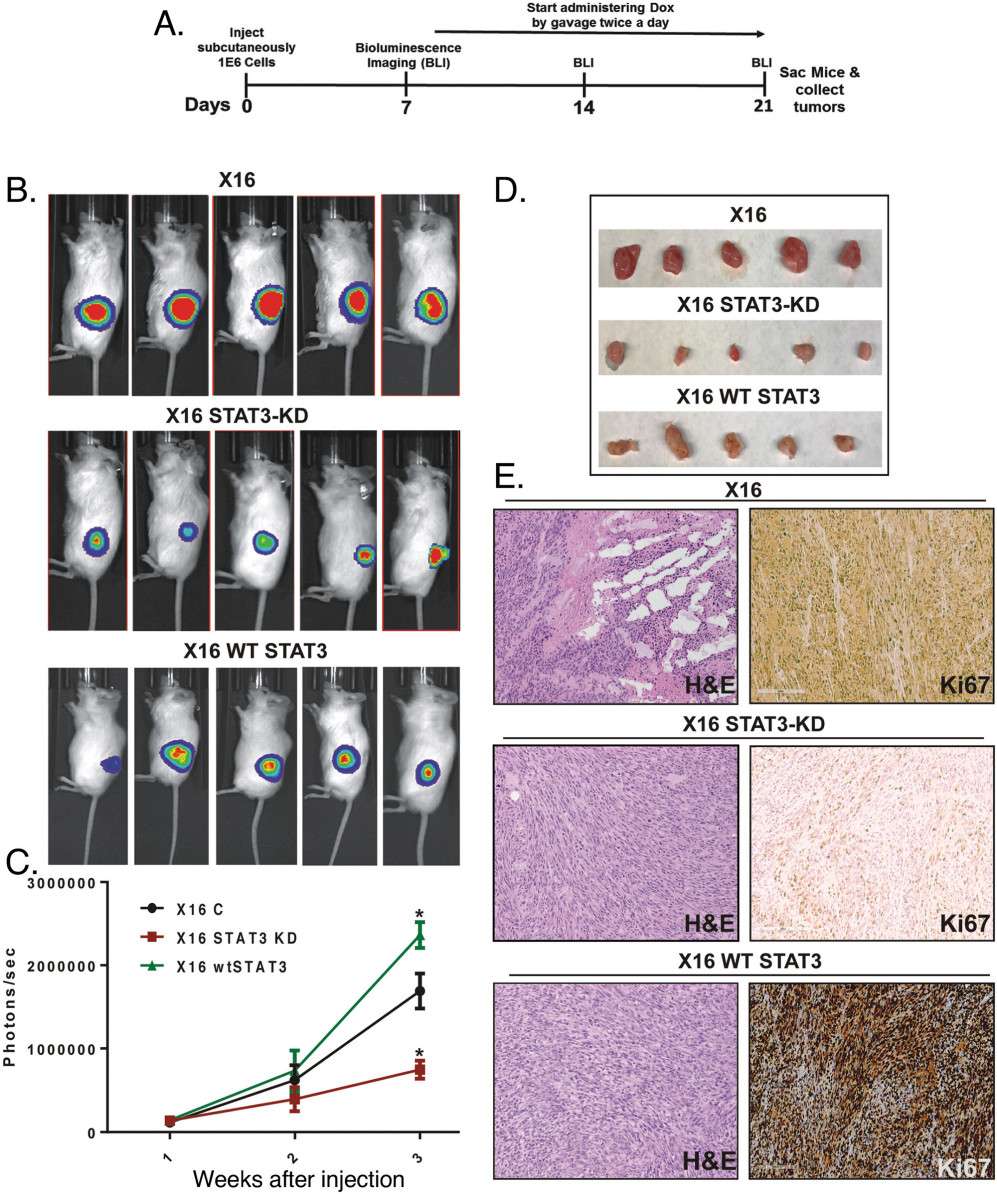
Effect of STAT3-KD on GBMX16 GIC tumorigenicity GBMX16 iSTAT3-KD GICs (10^6^ cells) transduced with luciferase-encoding lentivirus were injected into the flanks of NSG mice. After tumor induction was validated, Dox was delivered by oral gavage twice daily, and tumor development was monitored by bioluminescence imaging (BLI). **(A)** Schematic of xenograft mouse experiments. **(B)** Representative bioluminescent images of mice injected with X16 iSTAT3-KD cells or harboring WT-STAT3, and then treated with Dox (X16 STAT-KD or X16 WT-STAT3) or not Dox-Treated (X16) at 21 days post-injection. **(C)** Photographs of the tumors extracted from the mice on the day of the sacrifice. **(D)** Quantification of the bioluminescence signal detected at 1, 2 and 3 weeks post-injection. **(E)** H&E and Ki67 staining of tumor tissue at necropsy.

**Figure 5:**
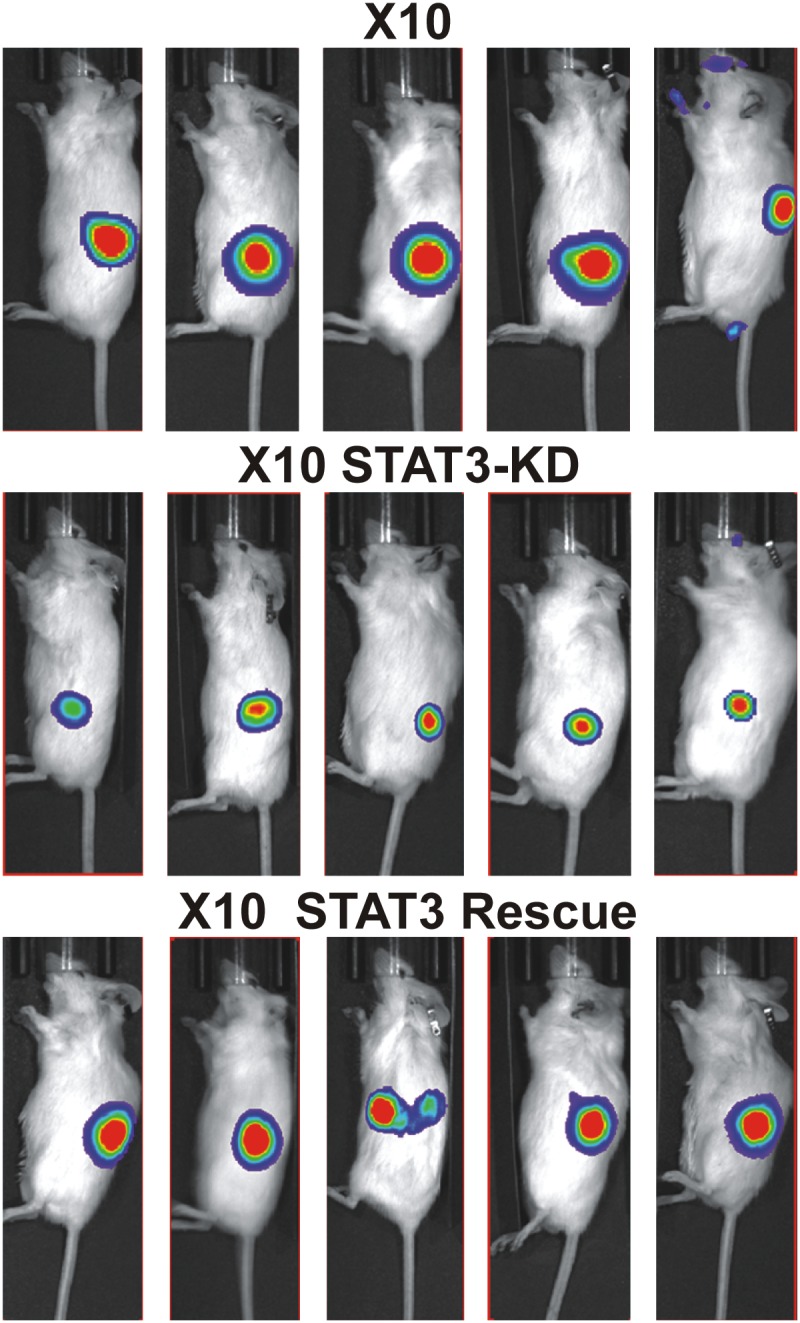
Effect of expression STAT3-KD and expression of WT- STAT3 on GBMX10 GIC tumorigenicity GBMX10 iSTAT3-KD GICs (10^6^ cells) transduced with luciferase-encoding lentivirus were injected into the flanks of NSG mice. After tumor induction was validated, Dox was delivered by oral gavage twice daily, and tumor development was monitored by live animal imaging. Representative bioluminescent images of mice injected with X10 iSTAT3-KD cells and then treated with Dox (X10 STAT3-KD) and rescued with WT-STAT3 (X10 STAT3 rescue) or not Dox-Treated (X10) at 21 days post-injection.

To investigate the role of STAT3 in GBM tumor progression, we examined the effect of Dox-inducible STAT3-KD on GIC tumorigenicity in NSG mice. Various combinations of oral gavage with Dox, Dox-containing chow and Dox-containing drinking water, did not markedly reduce STAT3 expression (<20%) in the iSTAT3-KD GICs injected orthotopically into the brains of NSG mice. Therefore, tumorigenesis studies were conducted as subcutaneous xenografts with Dox delivered by oral gavage twice a day. In brief, GBMX16 iSTAT3-KD GICs were transduced with luciferase-encoding lentivirus and injected into the flanks. After tumor induction was validated by live animal imaging (∼1-week post- injection), Dox treatment was begun, and tumor development was monitored for two weeks (Figure 4A). GIC-induced tumors grew rapidly over the course of the study, but tumor growth was significantly inhibited in Dox-treated mice injected with iSTAT3-KD GBMX16 GICs as evidenced by bioluminescence (Figure 4B). Furthermore, the tumors produced by STAT3-KD GBMX16 GICs grew slower and were much smaller (Figure 4C, 4D). As expected, WT-STAT3 expression in STAT3-KD GICs restored vibrant tumor growth in the mice (Figure 4B-4D), demonstrating that the difference in tumor growth was STAT3-dependent. Histopathology of the subcutaneous tumors produced by GBMX16, and STAT3-KD and WT-STAT3 rescued GICs showed that they were both high-grade astrocytomas. Staining of tumor tissue with Ki67, a marker for proliferating cells, revealed high numbers of proliferating cells in GBMX16 control and STAT3-rescued tumors as compared to the near absence of Ki67-positive cells in STAT3-KD tumors (Figure 4E). Similar to the findings with GBMX16 GICs, the tumorigenesis of GBMX10 GICs was also markedly reduced by STAT3-KD (Figure 5). The marked effect of STAT3-KD on the tumorigenicity of GBM X10 GICs was unexpected given that iSTAT3-KD only had a slight effect on GIC proliferation *in vitro* (Figure 3B).

### Effect of expression of STAT3 phosphorylation-defective mutants in STAT3-KD GICs on tumorigenicity

**Figure 6:**
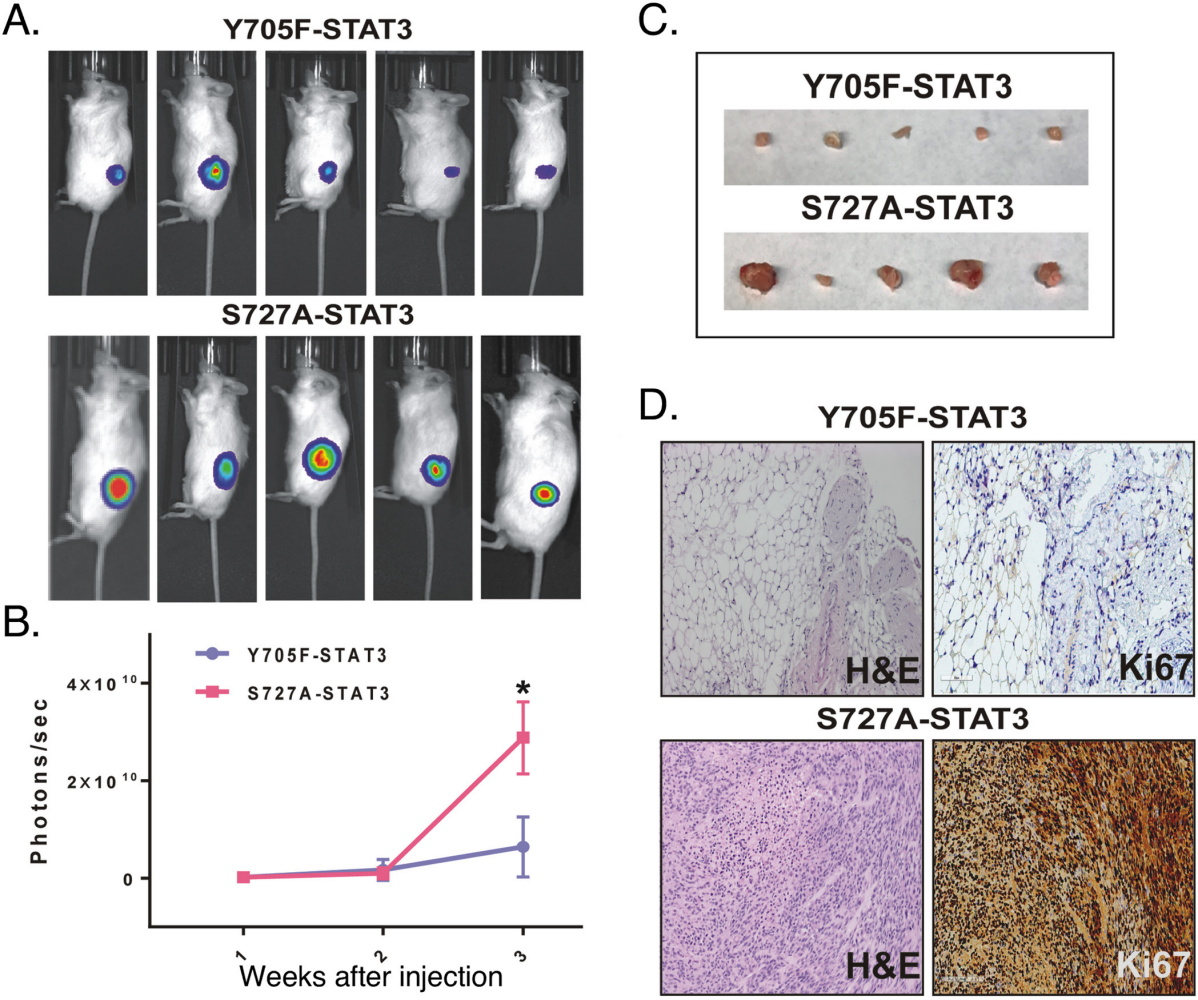
Effect of expression of STAT3 mutants on GBMX16 STAT3-KD GIC tumorigenicity Xenograft study was performed as in Figure 4 with GBMX16 iSTAT3-KD GICs rescued with Y705F-STAT3 or S727A-STAT3. **(A)** Representative bioluminescent images of mice at 21 days post-injection. **(B)** Quantification of the bioluminescence signal detected at 1, 2 and 3 weeks post-injection. **(C)** Photographs of the tumors extracted from the mice at necropsy. **(D)** H&E and Ki67 staining of tumor tissue at necropsy.

Since both the pY705 and pS727 sites of STAT3 regulated GIC function *in vitro*, we injected GBMX16 GICs harboring these STAT3 phosphorylation-defective mutants subcutaneously into the flanks of mice, and tumor growth was monitored by live animal imaging. Dox treatment was initiated at 1 week after confirming the induction of tumor growth. GBMX16 GICs harboring the Y705F-STAT3 mutant in STAT3-KD GICs produced very weak bioluminescence (Figure 6A). At necropsy, only small subcutaneous masses were found, and few tumor cells could be detected histologically (Figure 6C). In contrast, after a short initial delay the S727A-STAT3 mutant produced a strong bioluminescent signal, indicating rapid tumor growth and large tumor masses were produced (Figure 6B, 6C). Histopathological analysis of the tumor tissue revealed that the tumors were high-grade astrocytomas. To characterize the proliferation status within the tumor tissue, Ki67 staining was performed and showed that tumors produced in mice injected with S727A-STAT3 GICs were strongly Ki67 positive (Figure 6D). In contrast, Ki67 staining was nearly undetectable in tumors from GICs harboring the Y705F-STAT3 mutant (Figure 6D). The marked differences in the tumorigenic properties of the GBMX16 GICs harboring the different phosphorylation mutants is important, since both mutants were unable to restore GIC proliferation *in vitro* (Figure 3C).

### Identification of STAT3-regulated genes in GICs

**Figure 7:**
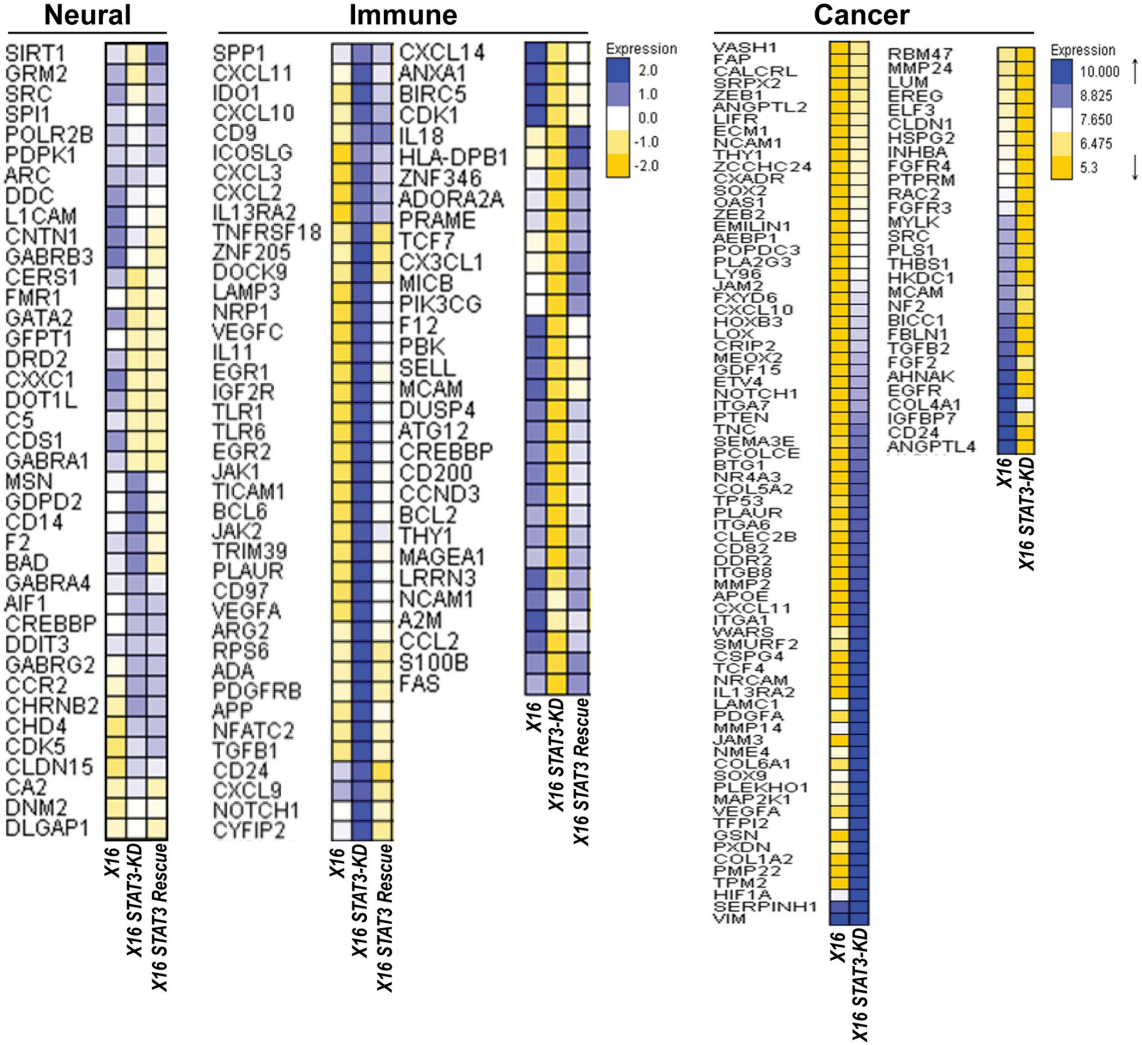
Effect of STAT3-KD on gene expression as determined by Nanostring array panels Total RNA was prepared from STAT3-KD and control GBMX16 GICs and gene expression profiling was conducted on the nCounter Analysis System using the PanCancer Progression, Neuropathology the PanCancer Immune Profiling Panels. Heat maps of STAT3-regulated genes are shown.

To identify genes and pathways regulated by STAT3 in GICs, we performed multiplex gene expression analysis using the nCounter PanCancer Progression, the Neuropathology and the PanCancer Immune Profiling Panels (Figure 7). STAT3-KD in GBMX16 GICs induced the expression of several classic IFN responsive genes, including the chemokine genes, *CXCL9, CXCL10* and *CXCL11.* Rescue with WT-STAT3 reduced the expression of these genes, indicating that STAT3 suppressed transcription of these classical IFN-responsive genes. In addition, STAT3-KD downregulated the expression of genes in GICs that are involved in cell proliferation (e.g., *CDK1, CDK2, CDK5, CDK5R1, CCND3* and *TCF7*), while inducing the expression of pro-apoptotic genes (e.g., *CASP6, CASP8, CDKN1A* and *ATG5*). WT-STAT3 restored the expression of most of these genes, suggesting that STAT3 promotes GIC proliferation and survival. These results are consistent with the findings that STAT3-KD in GBMX16 GICs reduced proliferation, while WT-STAT3 expression restored cell proliferation in STAT3-KD GICs. To validate our findings on STAT3 regulated genes in GICs, we determined the expression of a subset of the regulated genes by qPCR (Table 1). Various genes in neural (*AMIGO*, *ARRB2*, *ANG* and *DRD2*), and the immune response (*PBK, S100B, THY1, HMGB1, CDK2, HLADPB1, CD200 and CCL2)* pathways were reduced by STAT3-KD in GICs and their expression was restored upon STAT3 expression. In contrast, genes in neural (*CDS1* and *CHD4*) and cancer-related (*BTG1*, *COL6A1*, *PMP22* and *HIF1A*) pathways were increased by STAT3-KD, and their expression was reduced by STAT3 expression. In addition, we examined the effects of rescue with the phosphorylation defective STAT3 mutants in STAT3-KD GBMX16 GICs on the expression of STAT3-activated genes. While *DRD2* and *GATA2* appeared to be Y705-STAT3 dependent genes, expression of *CCL2*, *HLA-DPB1* and *PMP22* appeared to be independent of Y705-STAT3 phosphorylation in GBMX16 GICs (Table 2).

**Table 1:** The effects of STAT3-KD and rescue with WT-STAT3 on gene expression in GBMX16 GICs

Neuro Panel				
Gene	Alias	STAT3 KD^a^	Rescue^b^	Function
		Fold Change		
**AMIGO**	**ALI2, AMIGO-1**	1.58 ⇓	1.16 ⇑	Myelination
**ARRB2**	**ARB2, ARR2**	1.31 ⇓	1.33 ⇑	Neural Structure, Signaling
**ANG**	**ALS9, RNASE4**	3.51 ⇓	1.28 ⇑	Angiogenesis
**DRD2**	**D2DR, D2R**	2.42 ⇓	6.67 ⇑	Transmitter release
**GATA2**	**DCML, IMD21**	58.06 ⇓	46.9 ⇑	Angiogenesis, Chromatin Modification
**CDS1**	**CDS**	12.34 ⇑	3.3 ⇓	Metabolism, Signaling
**CHD4**	**Mi-2b, Mi2-beta**	1.51 ⇑	3.1 ⇓	Chromatin Modification

Immune Panel				
Gene	Alias	STAT3 KD	Rescue	Function
		Fold Change		
**PBK**	**SPK, CT84**	3.54 ⇓	2.06 ⇑	Tumorigenesis
**S100B**	**NEF**	1.36 ⇓	1.9 ⇑	Cell Cycle
**THY1**	**CD90**	3.5 ⇓	2 ⇑	Adhesion
**HMGB1**	**SBP-1**	2.15 ⇓	1.68 ⇑	Metastasis
**CDK1**	**CDC2**	2.51 ⇓	1.07 ⇑	Cell Cycle
**HLADPB1**	**DPB1**	1.25 ⇓	4.4 ⇑	Immunity
**CD200**	**OX-2, MOX1**	7.2 ⇓	3.74 ⇑	Immune suppression
**CCL2**	**MCAF, MCP1**	13.7 ⇓	6.25 ⇑	Inflammation and Immune regulation

Cancer Panel				
Gene	Alias	STAT3 KD	Rescue	Function
		Fold Change		
**BTG1**	**APRO2**	2.92 ⇑	5.91 ⇓	Anti-tumorigenic
**COL6A1**	**OPLL, BTHLM1**	6.54 ⇑	4.5 ⇓	Adhesion
**PMP22**	**CIDP, CMT1A**	1.82 ⇑	2.7 ⇓	Anti-proliferative
**HIF1A**	**HIF1-alpha**	1.46 ⇑	2.63 ⇓	Metabolism, Apoptosis, Angiogenesis

^a^ Fold change = gene expression in control GBMX16/STAT3-KD.

^b^ Fold change = gene expression in WT-STAT3 rescue/STAT3-KD.

**Table 2:** The effects of rescue with STAT3 phosphorylation deficient mutants on gene expression in GBMX16 GICs

Gene	Alias	Y705F	S727A	Function
		Fold Change		
**DRD2**	**D2DR, D2R**	4.2 ⇑	125.3 ⇑	Transmitter release
**GATA2**	**DCML, IMD21**	14.85 ⇑	80.2 ⇑	Angiogenesis, Chromatin Modification
**CD200**	**OX-2, MOX1**	2.24 ⇑	3.46 ⇑	Immune suppression
**CCL2**	**MCAF, MCP1**	7.24 ⇑	4.72 ⇑	Inflammation and Immune regulation
**HLADPB1**	**DPB1**	9.5 ⇑	5.6 ⇑	Immunity
**PMP22**	**CIDP, CMT1A**	56.6 ⇑	40.8 ⇑	Anti-proliferative

Fold change = gene expression in STAT3 mutant restored GICs/STAT3-KDs.

**Figure 8:**
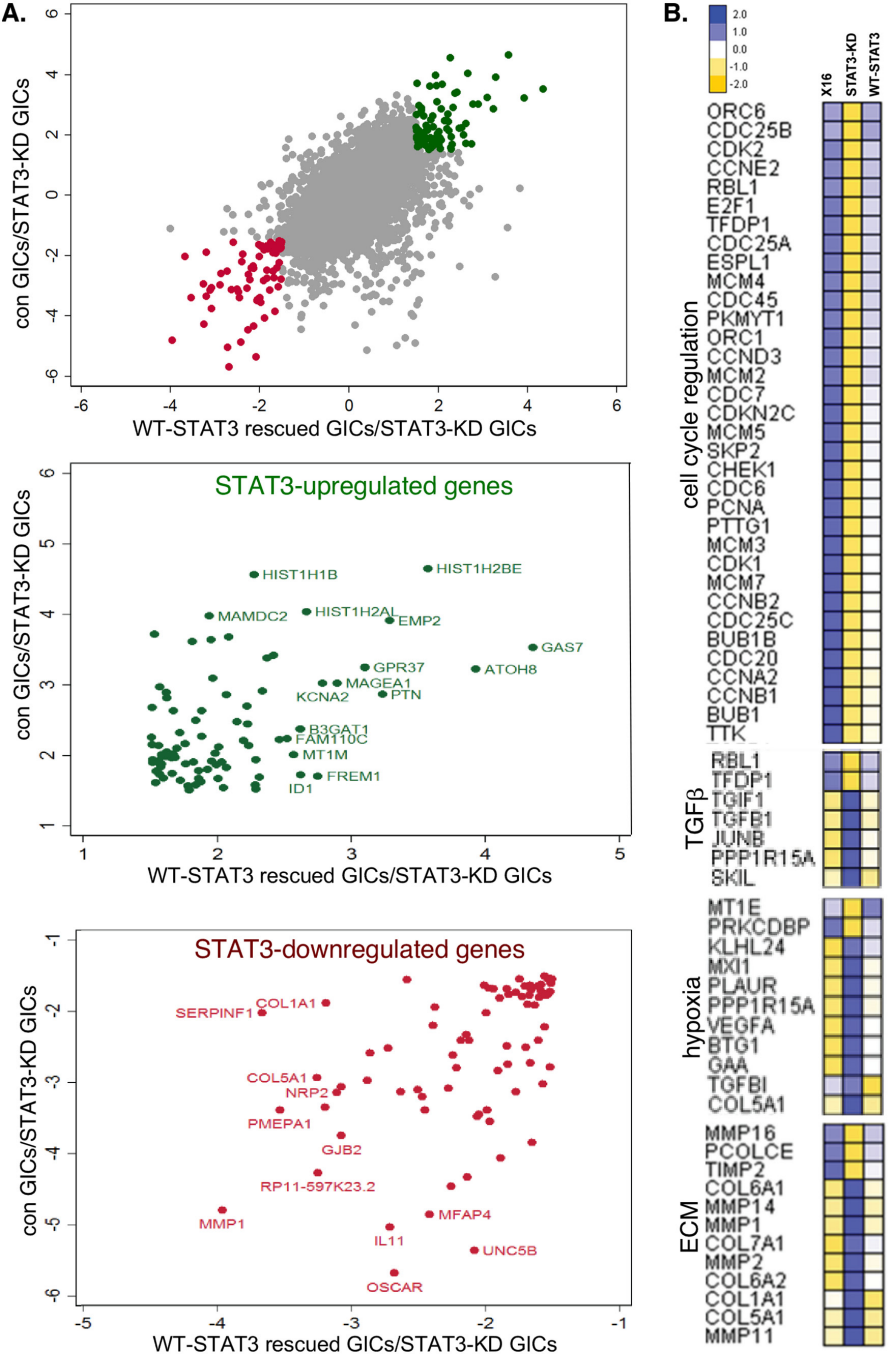
Effect of STAT3-KD on gene expression as determined by RNA-Seq RNA-Seq analysis was performed on RNA prepared from GBMX16 GICs not treated with Dox, GBMX16 GICs treated with Dox (STAT3-KD GICs), and WT-STAT3 rescued STAT3-KD GICs. **(A)** Concordance plot of genes positively (green) and negatively (red) regulated by STAT3 using a 1.5-fold cut-off. **(B)** Heat maps of STAT3-regulated genes involved in the cell cycle, TGFβ pathway, hypoxia response, and extracellular matrix.

As an unbiased approach for further defining the role of STAT3 in regulating gene expression in GICs, we performed RNA sequencing (RNA-seq) on RNA prepared from GBMX16 GICs not treated with Dox (con GICs), iSTAT3-KD GICs treated with Dox (STAT3-KD GICs), and iSTAT3-KD GICs treated with Dox and expressing WT-STAT3 (WT-STAT3 rescued GICs). To determine the concordance in STAT3 regulated genes, we plotted on the y-axis the fold-change in genes in GBMX16 GICs not treated with Dox versus STAT3-KD GICs, and on the x-axis the fold-change in genes in WT-STAT3 rescued GICs versus STAT3-KD GICs (Figure 8A). Using a 1.5-fold cut-off for the RNA-seq data, STAT3 did not regulate most transcripts in GICs under the conditions examined. However, ∼160 genes were concordantly upregulated (in green) or downregulated (in red) by STAT3. The function of the STAT3-regulated genes was then analyzed using the MsigDB [16] and Enrichr programs [17]. As shown in the heat maps, the STAT3-activated genes were over-represented by cell-cycle regulator genes (Figure 8B), which converge on CDK1 and CDK2 networks based on analysis of protein-protein interaction (PPI) and kinase-substrate networks (Supplementary Figure 3). Furthermore, many of the STAT3-regulated cell cycle genes were also identified by the targeted Nanostring arrays. In contrast, STAT3 appeared to selectively downregulate genes involved in the hypoxia response, the TGFβ pathway, and remodeling of the extracellular matrix (ECM) (Figure 8B). Furthermore, PPI and kinase-substrate network analysis of STAT3-downregulated genes in GICs suggests that STAT3 may suppress stress responses mediated by the TGFβ1/SMAD3 and mTOR/Protein kinase A pathways (Supplementary Figure 3).

**Figure 9:**
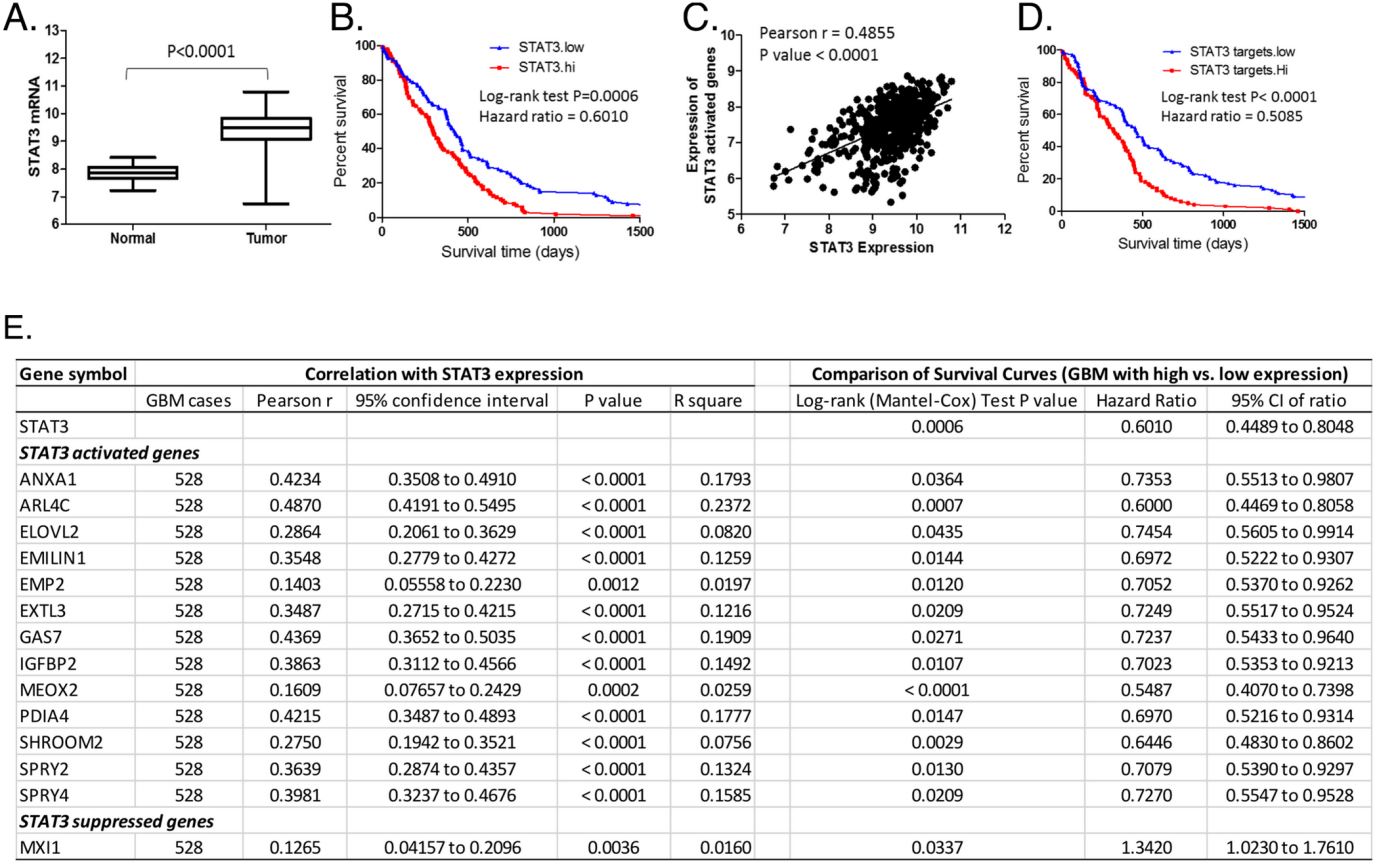
Identification of downstream effectors of STAT3 in GBM tumor specimens The expression of STAT3 and its targeted genes identified in the GBMX16 GICs by RNA-seq was examined in 528 GBM specimens included in the TCGA database. **(A)** Expression levels of STAT3 mRNA in GBM tumors and adjacent normal tissues. **(B)** Survival curves of GBM with high or low expression of STAT3. **(C)** Correlation between STAT3 expression and averaged expression level of STAT3-activated genes in GBM as listed in the inserted table. **(D)** Survival curves of GBM with high or low expression of STAT3-activated genes. **(E)** Outcome parameters from correlation analysis of the expression levels of STAT3-target genes and STAT3, and outcome parameters from survival curve comparison of GBM with high (top 25%) and low (bottom 25%) expression of STAT3 or its target genes.

In order to identify STAT3-targeted genes that are relevant to the clinical features of GBM, the expression of STAT3 and its target genes identified by RNAseq in the GBMX16 GICs were then examined in 528 GBM specimens included in The Cancer Genome Atlas (TCGA) database [8]. STAT3 expression was significantly increased in tumor tissues compared to the adjacent normal tissues (Figure 9A), and higher STAT3 expression in tumors was associated with shorter interval of survival of GBM patients (Figure 9B). Among the STAT3-activated genes, we identified 13 genes whose expression is correlated with STAT3 expression and associated with poor survival (Figure 9C-9E). Among the STAT3-suppressed genes, only *MXI1* showed an inverse correlation with STAT3 at the mRNA level in GBM and its higher expression was associated with longer survival (Figure 9E). These genes likely function as key downstream effectors of STAT3 in GBM cells.

## DISCUSSION

The identification of dysregulated signaling pathways in cancer is critical for the development of novel anticancer therapeutic strategies. Previous studies have implicated the STAT3 pathway as a key dysregulated pathway in cancer as evidenced by its constitutive tyrosine phosphorylation in glioma, as well as in breast, colon, kidney and endometrial cancers [10, 18–21]. To define the STAT3 pathway in GBM, we deleted the STAT3 gene in the established MT330 GBM cell line by CRISPR/Cas9 editing. Interestingly, STAT3-KO and STAT3-EV MT330 cells proliferated similarly *in vitro*. However, STAT3-KO MT330 cells failed to form intracranial tumors *in vivo*, probably through STAT3 affecting the tumor microenvironment. Rescue with WT-STAT3 restored tumorigenicity to that observed with EV-transduced MT330 cells, demonstrating that STAT3 was crucial for GBM tumorigenesis *in vivo* but STAT3 did not regulate GBM cell proliferation *in vitro*.

GICs isolated from PDXs are responsible for tumor initiation, propagation, recurrence and therapeutic resistance of GBM, and thus represent critical therapeutic targets [22]. We previously showed that STAT3 is phosphorylated at Y705 in GICs isolated from the GBM6, GBMX10, GBMX16 and GBMX39 PDXs, and that the JAK tyrosine kinase inhibitor WP1066 blocks Y705-STAT3 phosphorylation and inhibited subcutaneous tumor growth by GBM6 GICs [11]. In the present report we found that STAT3 also underwent phosphorylation at S727 in GICs isolated from these different PDXs. Interestingly, when GICs were induced to differentiate, reduction in both tyrosine and serine STAT3 phosphorylation was observed. Moreover, subcutaneous tumors formed by GICs grew more rapidly than tumors formed by the corresponding differentiating cells. These findings led us to investigate the role of the individual serine and tyrosine STAT3 phosphorylation sites in GICs. We attempted to knockout STAT3 in GICs by CRISPR/Cas9 gene editing but we were unable to isolate viable STAT3-KO GICs. In addition, using STAT3-specific shRNAs that successfully knocked down STAT3 expression in established MT330 cells, we were also unable to isolate GICs with constitutive STAT3-KD. However, GICs could be transduced with a Dox-inducible STAT3 knockdown construct (iSTAT3-KD), following which STAT3 expression was knocked down by ∼80% upon Dox treatment. Interestingly, while STAT3-KD significantly reduced the proliferation of GBMX16 GICs, STAT3-KD had only a negligible effect on the proliferation of GBMX10 GICs. Thus, the GBMX10 GICs resemble the established MT330 cell in that they may not require STAT3 for cell proliferation *in vitro*. However, STAT3-KD in GBMX10 GICs was not complete, because STAT3 signal could still be identified (Figure 3A).

To interrogate the functional roles of STAT3 phosphorylation sites in GICs, we expressed phosphorylation-defective STAT3 mutants in GICs already transduced with iSTAT3-KD construct and then treated GICs with Dox to knockdown endogenous STAT3 expression. We found that, while S727-STAT3 phosphorylation was dependent on Y705-STAT3 phosphorylation, STAT3-Y705 phosphorylation was independent of S727-STAT3 phosphorylation. This finding suggested that STAT3 phosphorylation occurs sequentially in the GICs, such that S727 phosphorylation occurred after Y705-STAT3 phosphorylation. Interestingly, in neural stem cells STAT3 reportedly undergoes S727 phosphorylation in the absence of Y705-STAT3 phosphorylation [23]. However, the sequential STAT3 phosphorylation events in GICs are reminiscent of cytokine-induced STAT3 activation, in which Y705-STAT3 phosphorylation preceded serine phosphorylation [24].

We then interrogated the function of these phosphorylation sites in GIC function *in vitro* and *in vivo*. Although expression of WT-STAT3 in STAT3-KD GBMX16 GICs rescued GIC proliferation, expression of the Y705F-STAT3 or S727A-STAT3 mutant did not rescue STAT3-dependent GIC proliferation. These results suggested that each STAT3 phosphorylation site independently regulates GIC proliferation. In addition, our data showed that STAT3 plays a critical role in GIC function *in vivo*. Tumors were induced in the flanks of immunocompromised mice instead of by intracranial injection, because Dox did not cross the blood-brain barrier, when STAT3-KD was induced by combining various routes of administration (water, food and oral gavage). We found that STAT3-KD in both GBMX16 and GBMX10 GICs markedly reduced tumor formation as compared to control GICs not treated with Dox. Rescue with WT-STAT3 restored GIC-induced tumorigenesis with tumors growing at a similar rate to tumors induced by GICs without STAT3-KD. Therefore, STAT3 not only stimulates GIC proliferation, as is the case in GBMX16 GICs, but also other GIC functions to promote GBM tumorigenesis. GBMX16 GICs harboring the S727A-STAT3 mutant produced tumors with a delay in onset, but these tumors were histologically similar to tumors without STAT3-KD. In contrast, GICs harboring the Y705F-STAT3 mutant failed to develop tumors, which demonstrated that Y705-STAT3 phosphorylation is critical for GIC tumorigenesis. Furthermore, since STAT3-KD GICs still produced tumors, our results indicate that the Y705F-STAT3 mutant has a dominant-negative activity in GIC tumorigenesis, which is in agreement with our finding that the Y705F-STAT3 mutant has dominant-negative activity in melanoma [25].

Since STAT3 was originally identified as a transcription factor in the acute-phase response [26, 27], we examined its role on GIC gene expression. We used both targeted cancer-related gene arrays and RNA-seq to investigate the role of STAT3 in gene regulation. Using cancer-related pathway probes, we showed that STAT3 inhibited the expression of several IFN response genes in GICs, such as the chemokines *CXCL9, CXCL10* and *CXCL11*, which is consistent with the finding that STAT3 can inhibit the expression of IFN response genes [28]. Since the IFN-response pathway can inhibit tumorigenesis through indirect effects on the tumor microenvironment [29], we propose that high STAT3 levels in GICs may inhibit these IFN-response genes and promote GBM tumorigenesis. We also found that STAT3 negatively regulated the expression of *BTG1, COL6A1, PMP22, HIFA, CDS1* and *CHD4*. However, STAT3 also promoted the expression of genes in neural pathways (*AMIGO*, *ARRB2*, *ANG* and *DRD2*), which may reflect the important role of STAT3 in promoting the neural stem cell phenotype in GICs. It is noteworthy that STAT3 increased DRD2 expression, since DRD2 has recently been found to promote GBM tumorigenesis and to be a potential important therapeutic target in GBM [30]. ANG is associated with blood vessel formation, and the association of STAT3 and angiogenesis has been previously reported [31]. We also found that *DRD2* and *GATA2* expression appeared be Y705-STAT3-dependent, which is consistent with Y705-STAT3 phosphorylation playing a critical role in GIC proliferation *in vitro* and tumorigenesis *in vivo*. However, we also showed that some STAT3-regulated genes, such as *HLA-DPB1* and *PMP22*, appeared to be independent of Y705-STAT3 phosphorylation in GBMX16 GICs (Table 2). Future studies are required to define the role that S727-STAT3 phosphorylation plays in regulating Y705-STAT3-independent gene expression, because in GICs S727-STAT3 phosphorylation requires Y705 phosphorylation.

By RNA-Seq analysis on the GBMX16 GICs we found that STAT3 regulated the expression of ∼160 genes associated with the cell cycle, the TGFβ signaling pathway, the hypoxia response and the ECM pathway. It is noteworthy that many of the STAT3-regulated genes identified in the targeted arrays were also identified by RNA-seq. Since the proliferation of GBMX16 GICs was STAT3 dependent, it is not surprising that STAT3 positively regulated the expression of several genes involved in cell cycle progression. However, our findings that STAT3-KD inhibits the tumorigenesis of GICs (GBMX10) but has only marginal effects on their proliferation *in vitro* highlights the importance of also uncovering STAT3-dependent pathways in GICs that may regulate tumorigenesis but are not *per se* required for GIC proliferation *in vitro*. RNA-seq showed that STAT3 negatively regulated genes involved in the TGFβ1 pathway, which can function as a tumor suppressor by directly inducing cell cycle arrest or apoptosis but also promotes the secretion of cytokines, growth factors, and extracellular matrix proteins to maintain cell and tissue homeostasis [32]. For example, STAT3 upregulated the expression of *MT1E*, the gene encoding metallotheonine-1E, which enhances human glioma cell migration and invasion by inducing the inactivation of *MMP-9* [33]. Interestingly, STAT3 also downregulated a number of members of the metalloprotease family which could act in a similar manner and promote tumorigenicity through increased GBM migration and invasion. STAT3 also increased *EMP2* expression, which has been recently found to promote angiogenesis in GBM [34]. STAT3 downregulated the expression of several genes that encode collagen subunits (*COL6A1, COL6A2, COL1A1, COL5A1, COL7A1*) that are components of the extracellular matrix, as well as matrix metalloproteases that breakdown the extracellular matrix (*MMP1, MMP2, MMP11, MMP14*). The low expression of these STAT3-regulated genes in GICs may reflect the critical role that STAT3 plays in the maintenance of stem cells. STAT3 also interacts with other transcription factors such as NFκB [24], and functions in the mitochondria to regulate cellular respiration [35]. Our findings demonstrate that STAT3 plays a critical role in GICs by not only regulating cell cycle progression but also cellular stress response.

By examining the expression of STAT3 and its target genes in GBM specimens included in TCGA, we found that upregulation of 13 STAT3-activated genes and downregulation of *MXI1*, a STAT3-suppressed gene, were associated with STAT3 activation and poor survival of GBM patients. These genes likely function as downstream effectors of STAT3 in GBM to regulate not only cell proliferation (e.g., *GAS7*, *IGFBP2*, *MEOX2* and *MXI1*), and but also tumor-stroma interactions by modulating protein secretion (e.g., *ANXA1*, *ARL4C*, *EMILIN1*, *EMP2*, *EXTL3* and *PDIA4*).

STAT3 is well-recognized as an oncogenic driver in cancer including GBM. STAT3 is activated by a large number of cytokines, chemokines and growth factors, many of which we found are also STAT3-regulated genes. Our data indicates that both Y705 and S727 phosphorylation are essential for GIC function *in vitro*, and Y705-STAT3 plays a highly critical role in GIC-driven tumorigenesis *in vivo*. We showed that STAT3 is sequentially phosphorylated first at Y705 and then at S727, and that S727 required Y705 phosphorylation. In GICs, STAT3 not only regulated pro-tumorigenic genes involved in cell cycle progression, remodeling of the extracellular matrix, as well as genes encoding cytokines and growth factors, while suppressing IFN response genes. Moreover, while some of the genes were dependent on Y705-STAT3 phosphorylation, other genes were independent of Y705-STAT3 phosphorylation. Taken together, our work reveals that GICs are highly addicted to STAT3, and STAT3 represents a promising molecular target.

## MATERIALS AND METHODS

### Cell culture

The MT330 (UTHSC Department of Neurosurgery) GBM cell line was grown in DMEM containing 10% fetal bovine serum (Atlanta Biologics) supplemented with penicillin (100 IU/ml) and streptomycin (100 μg/ml) at 37°C with 5% CO_2_. The GBM6, GBMX10, and GBMX16 patient-derived xenolines (PDXs) were maintained as xenografts in immunocompromised mice as described previously [15]. GICs were isolated and maintained in flasks precoated with poly-D-lysine and laminin, and grown in NeuroBasal-A medium (Invitrogen, Carlsbad, CA) containing 2% B27 supplement, 2 mM L-glutamine, 100 units/ml penicillin, 100 g/ml streptomycin, EGF (20 ng/ml), and basic FGF (40 ng/ml). MT330 human GBM cells were maintained in DMEM (Cellgro, Mediatech Inc., Manassas, VA) supplemented with 10% heat-inactivated fetal bovine serum (Hyclone, Logan, UT), 100 units/ml penicillin, and 100 g/ml streptomycin. GICs were induced to differentiate by culturing in differentiation medium (DMEM containing 10% fetal bovine serum), which was changed every third day.

### STAT3 knockout (KO) in MT330 cells

The STAT3 sgRNA iCRISPR lentiviral set for STAT3-KO was purchased from ABM (Richmond, Canada). A control lentiviral vector was constructed by inserting E-GFP gRNA sequences into pLenti CRISPR V2 lentiviral vector. All gRNA sequences were selected from the Human GeCKOv2 CRISPR knockout pooled library. Lentivirus were produced by packaging in 293FT cells as we published previously [36]. Stable pools of STAT3-KO MT330 cells were generated by transduction with the lentiviral CRIPSR/Cas9 vectors, selected with 5 μg/ml puromycin, and maintained without puromycin.

### Inducible STAT3 knockdown (iSTAT3-KD) in GICs and expression of STAT3 phosphorylation-defective mutants

To knockdown (KD) STAT3 expression, GICs were transduced with a Doxycycline (Dox)-inducible SMARTvector from GE Dharmacon (Lafayette, CO) harboring the inducible STAT3-KD shRNA with a turboRFP reporter, and flow sorted for RFP expression after Doxycycline hyclate (Dox) (MP Biomedicals, Santa Ana, CA) treatment to generate a pool of iSTAT3-KD GICs. Wild-type and mutant Y705F-STAT3 and S727A-STAT3 (Addgene, Cambridge, MA) constructs were cloned into a lentiviral vector with a bidirectional promoter driving expression of puromycin-resistance and E-GFP (System Biosciences, Palo Alto, CA). The STAT3 constructs were transduced into iSTAT3-KD GICs, and flow sorted for RFP and GFP positive cells to generate stable pools of cells expressing wild-type or mutant STAT3 constructs.

### Cell proliferation assays

GICs were seeded in 96-well plates (4,000 cells/well) and after ∼24 hr cells were treated with Dox. The number of viable GICs were measured using the Cell Titer glow luminescence viability assay (CellTiter-Glo, Promega) according to the manufacturer’s protocol with Dox replenished on alternate days.

### Gene expression analysis

Total RNA was extracted using the QIAshredder and RNeasy mini kits (Qiagen Inc., Frederick, MD) according to the manufacturer’s protocol. Gene expression was determined by quantitative real time PCR (qPCR) using gene-specific primers (listed on Supplementary Table 1) and an iScript one-step RT-PCR kit with SYBR Green (Bio-Rad). Reaction parameters were as follows: cDNA synthesis at 50 °C for 20 min, transcriptase inactivation at 95 °C for 5 min, and PCR cycling at 95°C for 10 sec and 60°C for 30 sec for 40 cycles.

### Immunoblotting

GICs were lysed at 4°C for 30 min in RIPA buffer (Sigma Aldrich, St Louis, MO) supplemented with protease inhibitor (Sigma Aldrich, St Louis, MO) and phosphatase inhibitor (BioTools, Jupiter, FL), followed by centrifugation (10,000xg for 10 min). Protein extracts (50μg) were separated by SDS-PAGE, transferred to polyvinylidene difluoride membranes (Millipore, Burlington, MA), and immunoblotted with the following antibodies: STAT3 and pS727-STAT3 (BD Biosciences, San Jose, CA), pY705-STAT3 (Abcam, Cambridge, A), STAT1 and STAT5 (Cell Signaling, Danvers, MA), and Actin (Santa Cruz Biotechnology, Dallas, TX). Following addition of IRDye800CW goat anti-mouse IgG or IRDye680 goat anti-rabbit IgG, blots were visualized on an Odyssey infrared imaging system (LICOR Biosciences, Lincoln, NE).

### Tumor xenografts in mice

All animal experiments were performed with at least 10 mice in each experimental group in accordance with a protocol approved by the Institutional Animal Care and Use Committee of the University of Tennessee Health Science Center. GICs were dissociated with HyQTase, resuspended in PBS, and enumerated in a cellometer (Nexelcom, Lawrence, MA). Heterotopic tumor xenografts were established in five-week-old male NOD.Cg *Prkdcscid Il2rgtm1Wjl*/SzJ (NSG) mice (Jackson Laboratory, Bar Harbor, ME) by injecting iSTAT3-KD GICs expressing luciferase (10^6^ cells /100μl PBS) into the flanks. After tumors were confirmed by live animal imaging following luciferin injection [37], Dox was delivered by oral gavage (10μg in 100μl of 0.9% saline) twice a day for two weeks. Tumor burden was assessed weekly by animal imaging and bioluminescence was analyzed with Living Image software (IVIS, Perkin Elmer, Waltham, MA) [37]. For orthotopic xenograft studies, luciferase-expressing MT330 cells (10^6^ cells /100μl PBS) were injected stereotactically into the superficial brain parenchyma of NSG mice through a burr hole in the skull [38], and tumor burden was assessed as described above. Tumor growth was analyzed using Graphpad Prism 7 software (La Jolla, CA). At 3 weeks following injection, animals were sacrificed and the tumors removed, fixed in 10% neutral buffered formalin, embedded in paraffin wax, sectioned, and subjected to hematoxylin and eosin (H&E) staining, and Ki67 (Abcam, Cambridge, MA) immunostaining.

### Nanostring gene expression analysis

Total RNA from GBMX16 cells was extracted using the QIAshredder and RNeasy mini kits (Qiagen Inc., Frederick, MD) according to the manufacturer’s protocol. Nanostring arrays were conducted against the PanCancer pathways, Immunology and the Neuropathology panels on the nCounter Analysis system (NanoString, Seattle, WA), and the data was analyzed with nSolver software using a 2-fold cutoff value as previously described [39].

### RNA sequencing

Total RNA was extracted using TRizol reagent (Ambion, Life Technologies) from control GBMX16 GICs or treated with Dox for 5 days, and RNA was resuspended in RNAse- and DNAse-free distilled water (Life Technologies). Sequences derived from total RNA paired end 100 bp sequences were mapped to the hg19 genome with the STRONGARM pipeline developed for the PCGP project [40] which employs bwa [41] and STAR [42] aligners. Transcript level data was counted using HTSEQ [43]. Transcript level log2FPKM (log2(FPKM +0.5)) was calculated and used to define log fold-change and average expression values. Transcripts with maximum global expression greater than 1 and log fold-change greater than 1.5 were selected for GO analysis in Enrichr [16]. Heat maps were visualized with Partek Genomics Suite 6.6 (St. Louis, MO) and scatterplots were visualized with STATA/MP 14.2 (College Station, TX).

### Data analysis

At least three independent experiments were performed in triplicate, and data are presented as mean ± SEM. Analysis of variance or Student’s t tests were performed and p ≤ 0.05 was considered to be statistically significant.

## SUPPLEMENTARY MATERIALS FIGURES AND TABLE


