# Supplementary material for: The critical role that STAT3 plays in glioma-initiating cells: STAT3 addiction in glioma
Source: Oncotarget. 2018 Apr 24;9(31):22095–112. doi: 10.18632/oncotarget.25188 (PMC5955139; doi:10.18632/oncotarget.25188)
Supplement: Supplementary file 1 [file oncotarget-09-22095-s001.pdf]

The critical role that STAT3 plays in glioma-initiating cells: STAT3 addiction in glioma

SUPPLEMENTARY MATERIALS

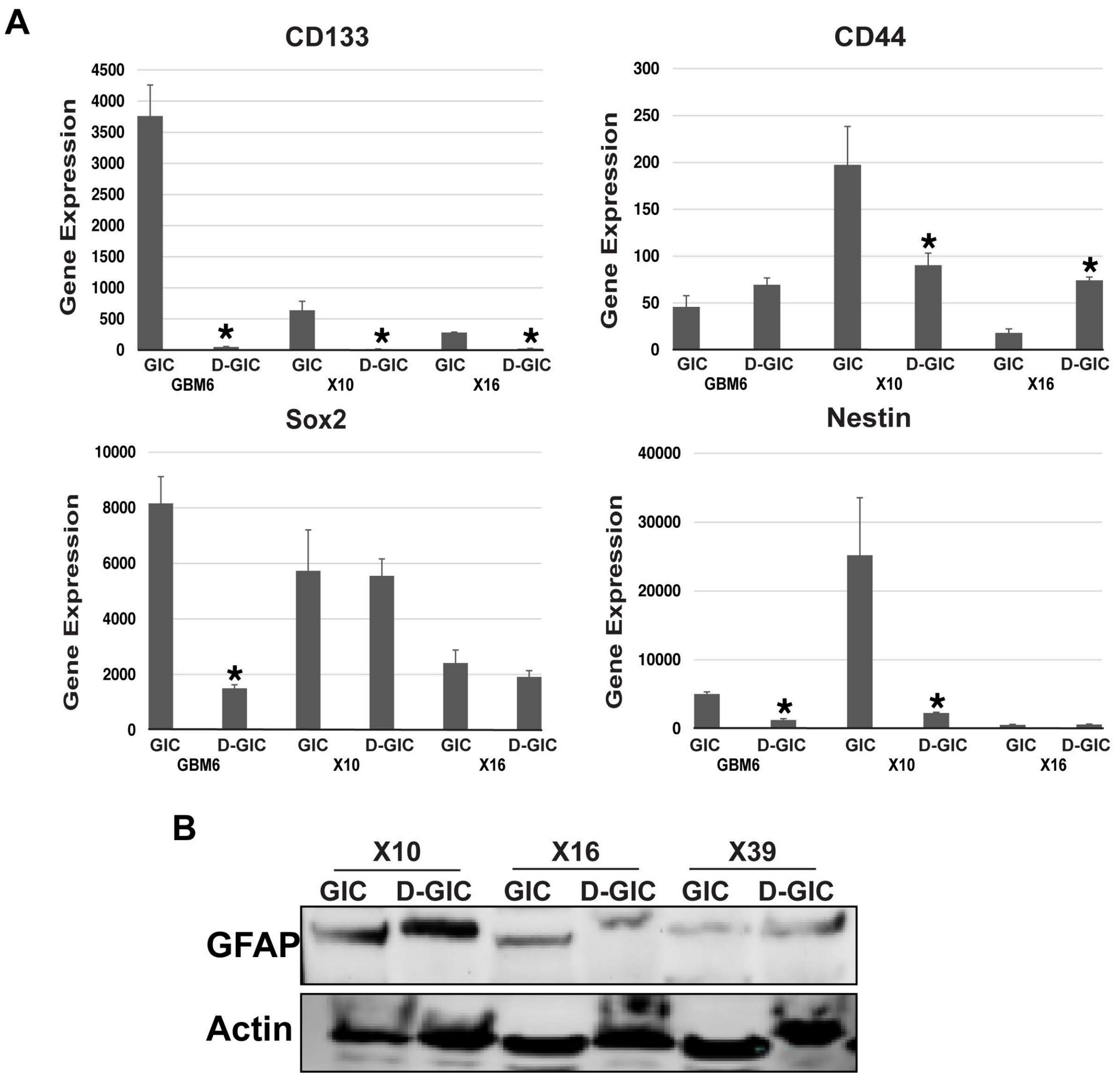

Supplementary Figure 1: Stem cell marker expression in the GICs and GICs induced to differentiate. GICs were grown under stem cell conditions or induced to differentiate for several passages in serum-containing medium (D-GICs). **(A)** qPCR was performed on RNA for *CD133*, *CD44*, *SOX2*, *NESTIN* and *Polr2A*, and gene expression normalized to Polr2A expression. **(B)** Protein lysates were immunoblotted for the astrocyte differentiation marker, GFAP, in the GBMX10, GBMX16 and GBX39 GICs and corresponding D-GICs.

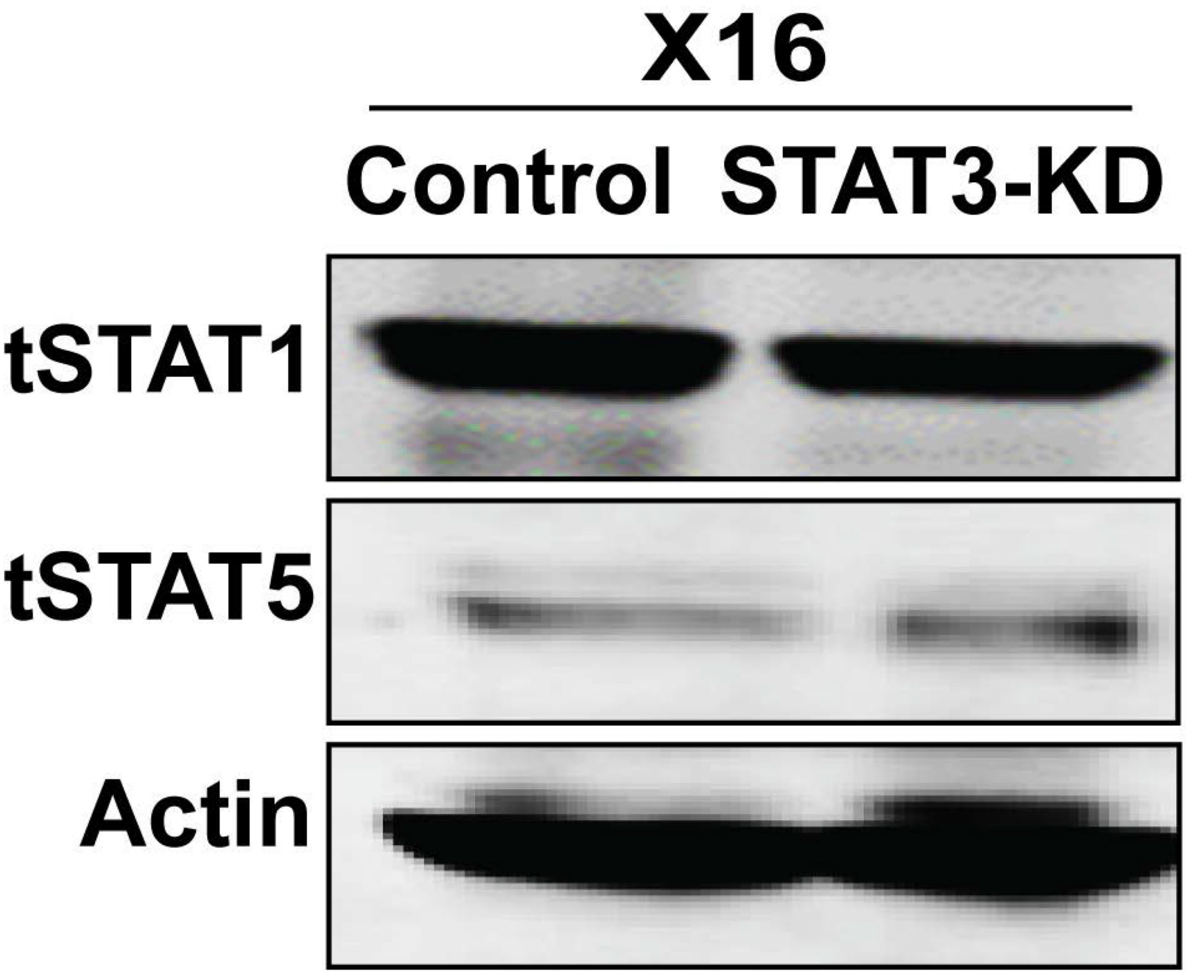

Supplementary Figure 2: STAT3-KD has no effect on STAT1 and STAT5 protein expression in the X16 GICs. Immunoblots of protein lysates from iSTAT3-KD GBMX16 GICs treated with Dox using antibodies against STAT1 and STAT5.

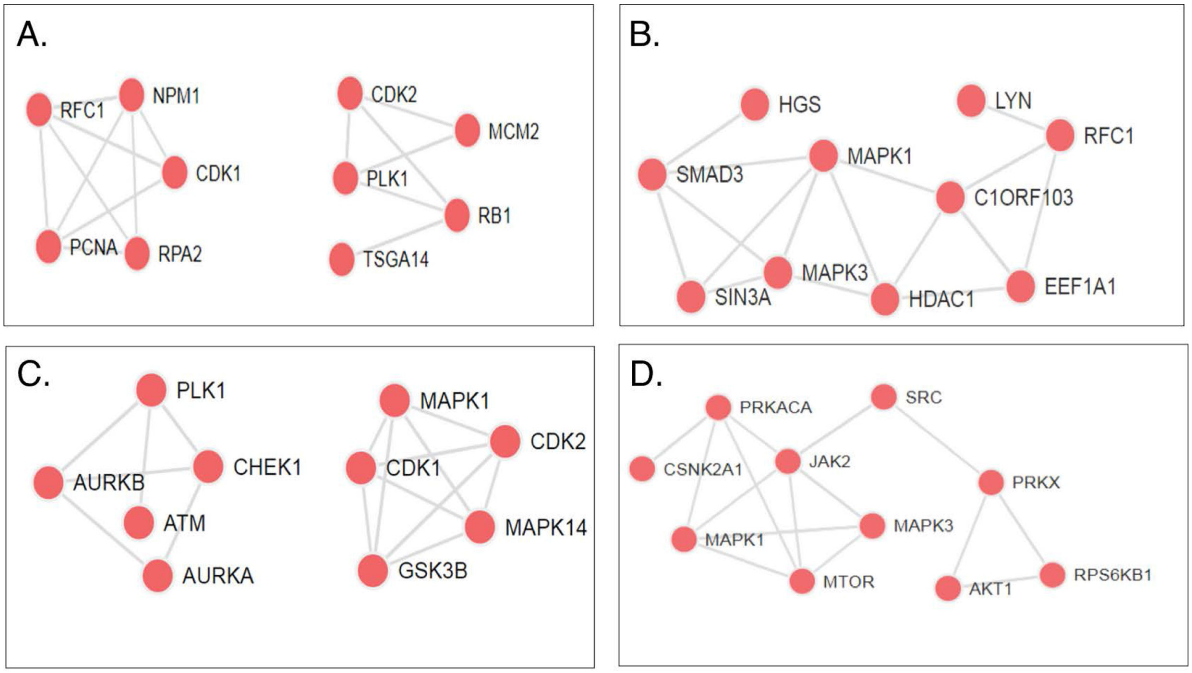

Supplementary Figure 3: Protein-protein interaction and kinase substrate networks analysis of STAT3-regulated genes identified by RNA-seq. RNA-Seq analysis was performed on RNA prepared from GBMX16 GICs not treated with Dox, GBMX16 GICs treated with Dox (STAT3-KD GICs), and WT-STAT3 rescued STAT3-KD GICs. **(A)** Protein-protein interaction (PPI)-Hub proteins associated with STAT3 activated genes. **(B)** Kinase substrate networks associated with STAT3 activated genes. **(C)** PPI-Hub proteins associated with STAT3 suppressed genes. **(D)** Kinase substrate networks associated with STAT3 suppressed genes.

Supplementary Table 1: Sequences of primers used for qPCR

Target	Sequence (5'-3')
POLR2A	5'ATGTCTGTGACGGAGGGTGGCA<br>3'GGCCAGGACACTCTGTCATGTTT
STAT3	5'CAGCAGCTTGACACACGGTA<br>3'GCCCAATCTTGACTCTCAATCC
CD133	5'AGTCGGAAACTGGCAGATAGC<br>3'GGTAGTGTTGTACTGGGCCAAT
CD44	5'TTACAGCCTCAGCAGAGCAC<br>3'TGACCTAAGACGGAGGGAGG
SOX2	5'GCCGAGTGGAAACTTTTGTCG<br>3'GGCAGCGTGTACTTATCCTTCT
NESTIN	5'CTGCTACCCTTGAGACACCTG<br>3'GGGCTCTGATCTCTGCATCTAC
AMIGO	5'TTCCGTACCCCACCAGAACA<br>3'AGCTCTGGCCACCTCAAAAA
ARRB2	5'CCATGGGGGAGAAACCCG<br>3'GGTAGTCAGGGTCCACAAGC
ANG	5'TTTCCATTGTCCTGCCCGTT<br>3'TTGGCATCATAGTGCTGGGT
DRD2	5'GCTGCTGAAAACCATCTGGC<br>3'GGTCCTGGAAGGTGACTCGT
GATA2	5'ACTCATCAAGCCCAAGCGAA<br>3'CTTCATGGTCAGTGGCCTGT
CDS1	5'TGTTTGGATTCATTGCTGCCTAT<br>3'TGGATCTGGAAAGGGTACAAGC
CHD4	5'CCAATGCAGTCCTGCACAAA<br>3'CTCACAGCAACTGGGGGAAT
PBK	5'GCGGTGAGACTCTGGACTGA<br>3'CTGCATAAACGGAGAGGCCG
S100B	5'TGTAGACCCTAACCCGGAGG<br>3'TGCATGGATGAGGAACGCAT
THY1	5'GAAGACCCCAGTCCAGATCCA<br>3'TATTCTCATGGCGGCAGTCC
HMGB1	5'CAGAGCGGAGAGAGTGAGGA<br>3'TTGCCTCTCGGCTTCTTAGG
CDK1	5'CTTGGCTTCAAAGCTGGCTC<br>3'GGGTATGGTAGATCCCGGCT
HLA-DPB1	5'GTCGTGTCCACCAACCTGAT<br>3'ACTCCACGGTGACAGGACTA
CD200	5'CACCGTCTATGTACAGCCCA<br>3'CTGGTAACAGACGTGGTCCC
CCL2	5'ACCTGGACAAGCAAACCCAA<br>3'AGGGTGTCTGGGGAAAGCTA
BTG1	5'CGCAACACATGGACTACCCT<br>3'CAGTAGTCAGCACTGGGGTG
COL6A1	5'ATTGCCAAGGACTTCGTCGT<br>3'TCCACTGCAGGCTCTTGATG
PMP22	5'TGGAAGAAGGGGTTACGCTG<br>3'TCTGCCAGAGATCAGTTGCG
HIF1A F1	5'GAAAGCGCAAGTCTTCAAAG<br>3'TGGGTAGGAGATGGAGATGC
